# Leptospirosis Cases During the 2024 Catastrophic Flood in Rio Grande Do Sul, Brazil

**DOI:** 10.3390/pathogens14040393

**Published:** 2025-04-17

**Authors:** Tani Maria Ranieri, Eduardo Viegas da Silva, Marcelo Jostmeier Vallandro, Mayara Mota de Oliveira, Regina Bones Barcellos, Roberta Vanacor Lenhardt, Loeci Natalina Timm, Aline Scarpellini Campos, Cintia Simoni, Paulo Renato da Silva Abbad, Doris Bercht Brack, Tássia Flores Rech, Juliano de Oliveira Silveira, Vivian Oliveira Estevam, Lidsy Ximenes Fonseca, Deise I. Galan, Maria Cristina Schneider

**Affiliations:** 1 State Health Surveillance Center, Health Department of the Rio Grande do Sul State, Porto Alegre 90610-000, Brazil; cevs-direcao@saude.rs.gov.br (T.M.R.); marcelo-vallandro@saude.rs.gov.br (M.J.V.); mayara-oliveira@saude.rs.gov.br (M.M.d.O.); regina-barcellos@saude.rs.gov.br (R.B.B.); roberta-lenhardt@saude.rs.gov.br (R.V.L.); loeci-timm@saude.rs.gov.br (L.N.T.); aline-campos@saude.rs.gov.br (A.S.C.); cintia-simoni@saude.rs.gov.br (C.S.); paulo-abbad@saude.rs.gov.br (P.R.d.S.A.); doris-brack@saude.rs.gov.br (D.B.B.); tassia-rech@saude.rs.gov.br (T.F.R.); juliano-silveira@saude.rs.gov.br (J.d.O.S.); vivian-estevam@saude.rs.gov.br (V.O.E.); 2 National Health Surveillance and Environmental Secretary, Ministry of Health of Brazil, Brasilia 70723-040, Brazil; lidsy.fonseca@saude.gov.br; 3 Department of Global Health, School of Health, Georgetown University, 3700 Reservoir Rd NW, Washington, DC 20007, USA; dgl32@georgetown.edu (D.I.G.); mcs368@georgetown.edu (M.C.S.)

**Keywords:** outbreak, leptospirosis, disaster, flood, Brazil

## Abstract

Leptospirosis is a well-known disease that frequently occurs after floods. At the beginning of May 2024, a catastrophic flood occurred in the state of Rio Grande do Sul, Brazil, affecting two million people and leading to a state of calamity. Given the State’s high pre-flood leptospirosis rates, the disease became a major concern for decision-makers. This study aimed to estimate the rise in leptospirosis cases after the flood, assess the changes in case patterns before and after the disaster, and document the response of the state health surveillance center to the outbreak. We estimated the increase during the flood period (May to July 2024) by comparing it with the same period in the previous year as baseline. During the first three months of the catastrophic event, 6273 suspected cases of leptospirosis were reported to the surveillance system, and 958 cases were confirmed. The number of confirmed cases during the flood was 10.3-fold higher than the baseline. Spearman’s rank-order correlation coefficient was 0.77 (*p*-value < 0.0001) for the association of Health Regions regarding the rank in incidence rates of confirmed cases in the flood period and the proportion of the population with households flooded. Thirty deaths (three females) were confirmed, a 6.0-fold rise compared with 2023. The state responders carried out many activities, including epidemiological surveillance and the dissemination of information. Among the challenges faced was the lack of evidence in the literature supporting recommendations for antibiotic chemoprophylaxis for rescue personnel. Another difficulty concerned performing gold-standard laboratory diagnostic tests to confirm the enormous number of suspected cases reported during this catastrophe. Despite implementing many actions to mitigate its impact, leptospirosis remained a major challenge during the event. These findings may provide valuable insights for decision-makers facing similar situations in massive climate disasters.

## 1. Introduction

Leptospirosis is a significant infectious disease that poses a serious public health threat, with frequent outbreaks following floods. Despite its impact, there have not been many advances in research to develop tools to save lives from this zoonosis. The disease is estimated to cause approximately one million human cases annually, with around 60,000 deaths worldwide, with the highest burden in tropical and subtropical countries [1,2]. Human infection primarily occurs through contact with water, mud, or soil contaminated with the urine of animals shedding the bacteria [2,3]. Increased leptospirosis cases have been documented after heavy rains and floods in tropical countries, including Brazil [4,5,6,7,8,9,10].

On 28 April 2024, the southern Brazilian state of Rio Grande do Sul experienced a meteorological event characterized by intense precipitation and high-velocity winds, culminating in record-breaking rainfall accumulation. In early May 2024, a prolonged period of sustained heavy rainfall triggered widespread hydrologic disturbances, including extensive inundation and geomorphological instability with landslides. These events resulted in significant infrastructural damage and the forced displacement of a substantial population. Consequently, both the state and federal governments of Brazil formally declared a state of public calamity in Rio Grande do Sul. Many municipalities also declared a state of emergency [11,12,13].

The state government established a Situation Room in the capital, Porto Alegre, with participation from various government secretariats and external organizations involved in disaster management. Additionally, the National Emergency Operations Center was set up to coordinate federal actions in partnership with the state and municipal levels of the Unified Health System (known by its Portuguese acronym, SUS). This disaster was considered the largest flood in the state’s history. Thousands of rescue personnel, both military and civilian, worked tirelessly to save people and animals. Many cities became inaccessible due to flooding and landslides, which destroyed roads and collapsed bridges.

Brazil has a decentralized universal public healthcare system (SUS) that serves approximately 75% of the population [14]. This system provides free access to medical appointments, hospitalizations, a wide range of medicines, vaccination campaigns, and preventive health surveillance. Leptospirosis is a disease subject to mandatory notification through Brazil’s Notifiable Diseases Information System (SINAN) [15]. Around 4000 confirmed leptospirosis cases are reported annually nationwide through the official surveillance system [16,17,18].

Leptospirosis incidence rates in Brazil are estimated at 1.9 cases per 100,000 inhabitants, with variations across different regions [17]. Floods are among the most frequently reported exposure factors in both urban and rural cases, particularly in areas with high rodent activity. In rural regions, additional risk factors include animal farming and agriculture, especially in the southern region of Brazil [17].

Rio Grande do Sul experiences a substantial burden of leptospirosis, with approximately 500 cases reported annually. The state’s rural areas bear a disproportionate share of this burden, with incidence rates of 11.0 per 100,000 inhabitants, three times higher than urban areas. While urban areas report a higher absolute number of cases, the stark disparity in incidence highlights the vulnerability of rural populations [17]. Previous studies have pinpointed high-risk areas within the state, particularly in the vicinity of the Jacuí and Taquari rivers, major lagoon systems, and the capital city [19,20,21]. These identified areas have documented clusters of leptospirosis cases [17]. These studies have also examined environmental factors influencing the distribution of leptospirosis cases, such as ecoregions, soil types, and land use patterns linked to economic activities [20,21,22]. These previous reports underscore the importance of a One Health approach in understanding and preventing leptospirosis outbreaks in Rio Grande do Sul, particularly given the persistence of these environmental drivers [23,24].

Leptospirosis presents a broad spectrum of clinical manifestations, ranging from mild illness to severe, potentially fatal cases. The global case–fatality rate varies between 5% and 30%, with Brazil reporting an average of 9% [25]. Early diagnosis is crucial in preventing severe cases and hospitalization, but laboratory testing for leptospirosis is complex, requiring different methods depending on the stage of the illness [3,25,26,27]. Currently, no human vaccine for leptospirosis is available in Brazil; only a few countries offer vaccines, generally for specific high-risk groups [24,28,29]. These challenges make managing leptospirosis outbreaks after floods even more difficult.

Following the catastrophic flooding in Rio Grande do Sul, leptospirosis emerged as a major concern for public health authorities. Exposure occurs both during the flood—affecting the general population and rescue personnel—and afterward, as receding waters leave contaminated mud inside and around homes, increasing exposure risk during cleanup efforts.

As responders, we faced numerous challenges in saving lives from leptospirosis during the flood. One major obstacle was the lack of accessible information to support decision-making. Significant gaps were identified in the literature regarding case prevention, including the use of chemoprophylaxis, estimates of expected cases and deaths, strategies for early case detection, and documentation of how other government agencies had responded to similar catastrophic events.

This study aimed to estimate the rise in leptospirosis cases after the flood, examine whether there were differences in the patterns before and after the disaster, demonstrate the correlation between leptospirosis cases and the flood, and document the response of the state health surveillance center to the outbreak. These findings may provide valuable insights for other areas facing similar challenges.

## 2. Materials and Methods

### 2.1. Study Area and the Event

Rio Grande do Sul is the southernmost state in Brazil, bordering Argentina and Uruguay. It covers an area of 281,707,151 km^2^, with 497 municipalities (second subnational level). The state is divided into seven Macroregions and thirty Health Regions (Figure S1), averaging 17 municipalities per Health Region [30,31].

In 2022, the population was 10,882,965 inhabitants, with 85.1% living in urban areas; 15% resided in the capital city of the Porto Alegre metropolitan area [30,32]. The State has one of the highest quality-of-life indices in the country, with a life expectancy at birth of 76.4 years and a literacy rate above 97%. In 2022, Rio Grande do Sul’s GDP per capita was approximately BRL 51,000/year, ranking fifth among Brazil’s 27 states in terms of contribution to the national Gross Domestic Product [31].

The state of Rio Grande do Sul has six ecoregions, the largest being the Uruguayan savanna (64%), followed by two types of forests in the central and northern areas (35%). The average monthly temperature is 18.85 °C, the precipitation of the wettest month is 173.1 mm, and the state average altitude is 352 m above sea level [21]. The state’s hydrology is divided into two main areas. One is primarily shaped by the Guaíba Basin, which includes major tributaries such as the Jacuí, Taquari, Cai, Gravataí, and Sinos rivers. These rivers are in the central part of the state, flowing through its most populated areas, including the capital city, Porto Alegre, and are historically prone to flooding. Another tributary flows into the Dos Patos Coastal Lagoon in the southern region. Much of the state is dedicated to agribusiness, including rice plantations [21].

The 2024 catastrophic flood event was considered the biggest flood documented in Rio Grande do Sul and required a large-scale response. On 28 May, according to Civil Defense [33], 471 of the 497 municipalities were affected. Approximately 2,345,400 people (21% of the state population) were impacted, 876,200 had their homes flooded [34,35], and 581,638 were displaced. During this period, 77,712 people and 12,521 animals were rescued by official staff (according to Civil Defense, 28,181 were on duty during the response) and by thousands of civilian volunteers. There were 183 confirmed deaths, and 27 people were reported missing [33,34,35,36]. By the end of May, 48,789 people were living in shelters (many with pets), set up in stadiums, schools, social clubs, churches, and other places throughout the state. The flood caused severe damage to crucial civilian and government infrastructure, including homes, schools, hospitals, and water and power systems. Nearly one million people lost access to drinking water, and food shortages persisted for days due to blocked roads and hampered supply deliveries [13]. Patients in affected hospitals had to be evacuated by helicopter or boat, and many healthcare facilities were either underwater or inaccessible. The main state airport, located in the capital, was submerged and took nearly six months to recover.

The State Health Surveillance Center (in Portuguese, Centro Estadual de Vigilância em Saúde; acronym, CEVS) under the Rio Grande do Sul Health Department was part of the emergency response, participating in the state’s Crisis Situation Room and the national-level Emergency Operations Center (EOC) [37].

### 2.2. Study Design and Data Collection

We conducted a retrospective epidemiological study in two parts, with the following aims: (1) to describe leptospirosis cases and deaths after the flood period by time and space, estimate the rise in leptospirosis cases during the event compared with the same period in the previous year as a baseline, and examine whether there were differences in the demographic characteristics of the cases after the flood compared with the baseline period and (2) to describe the response of CEVS, including the challenges faced.

The study included all municipalities in the state of Rio Grande do Sul, aggregated by Health Regions and focused on the first three months following the flood (1 May to 31 July 2024). To estimate the increase in leptospirosis cases and identify changes in case patterns, we compared the flood period with the same timeframe in the previous year (1 May to 31 July 2023), which served as a baseline representing the endemic period. This baseline was chosen due to the significant underreporting of leptospirosis cases during the COVID-19 pandemic (2020–2022).

All information used in this study was provided by CEVS. Case notifications were submitted by each municipality through the National Information System of Notifiable Diseases (in Portuguese, Sistema de Informação de Agravos de Notificação—SINAN, accessed on 13 March 2025).

According to the Surveillance Guidelines from Brazil’s Ministry of Health [38,39], a leptospirosis case can be confirmed by clinical–laboratory or clinical–epidemiological criteria. During the flood period, diagnostic tests used to confirm cases at the Rio Grande do Sul Central Laboratory included the IgM antibody test (IgM) and real-time polymerase chain reaction (qPCR). The microscopic agglutination test (MAT) was performed by the National Reference Laboratory for Leptospirosis (LRNL) at Fiocruz IOC/RJ. The criteria for leptospirosis case definitions, as outlined by Brazil’s Ministry of Health, along with methodological details—including MAT cut-off titers, MAT serovars, the ELISA kit manufacturer and type, the diagnostic sensitivity and specificity, and the qPCR method—are provided in Supplemental Material Tables S1 and S2 [38]. The guidelines also provide instructions for healthcare providers on identifying symptoms and conducting laboratory tests specific to the different phases of leptospirosis.

Population data for incidence rate calculations were obtained from the Brazilian Institute of Geography and Statistics (Portuguese acronym: IBGE) [30,32]. We calculated the incidence per 100,000 inhabitants by health region and demographic characteristics. The study used data from the latest Brazilian Census, conducted in 2022. The percentage of the population with flooded households in municipalities under a state of public calamity was also obtained from the Institute of Applied Economic Research—IPEA [34]. This variable was used to assess the correlation between the incidence rate of confirmed cases and the proportion of the population at higher risk of leptospirosis due to the flood at the Health Region level.

Confirmed leptospirosis cases were described by time (epidemiological weeks), space (Health Regions), and demographic characteristics (age and sex), comparing the flood period to the baseline. The Health Regions were grouped into quintiles (five groups representing 20% of the data) to facilitate the description. Spearman’s rank correlation coefficient (rho) was used to assess the level of correlation between the incidence of confirmed cases in Health Regions during the flood period (May to July 2024) and the percentage of the Health Region population with flooded households during the event. Chi-squared tests of heterogeneity were used to examine differences between the demographic characteristics of confirmed cases in the flood and baseline periods.

To estimate the rise in leptospirosis cases and deaths after the flood in each Health Region and across the entire state, we compared the total number of confirmed cases during the flood period with the corresponding period in the previous year. Maps were created using QGIS 3.34, and statistical analyses were conducted using Stata 15.1. Associations with *p*-values less than 0.05 were considered statistically significant.

## 3. Results

### 3.1. Leptospirosis Cases and Deaths During the Flood Period Compared with the Previous Year

#### 3.1.1. Leptospirosis Notified and Confirmed Cases

During the catastrophic flood period analyzed in this study (1 May to 31 July 2024), 6273 suspected cases of leptospirosis were reported to the state health surveillance center, of which 958 (15.3%) were confirmed by different diagnostic methods, including 464 confirmed by clinical–laboratory criteria. During the same period in the previous year, 461 suspected cases were reported, with 93 confirmed (20.2%), including 73 by clinical–laboratory criteria.

**Figure 1:**
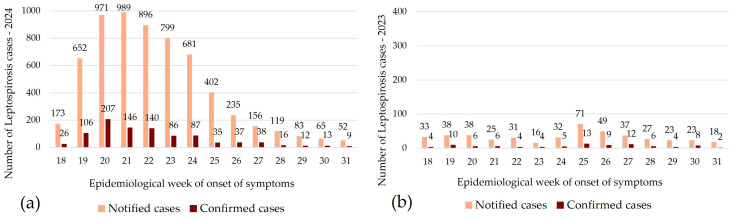
Distribution of leptospirosis cases notified and confirmed by epidemiological week of onset of symptoms (**a**) during the flood period (1 May to 31 July 2024) and (**b**) during the same period in the previous year (1 May to 31 July 2023), Rio Grande do Sul, Brazil. The scale in (**a**) (0 to 1000) is 2.5 times higher than the scale in (**b**) (0 to 400).

During epidemiological weeks 20 to 22, corresponding to two and three weeks after the flood began, a peak in the number of suspected and confirmed cases (considering the week of symptom onset) was observed. During these three weeks, an average of 952 suspected cases were reported per week (Figure 1a), compared to 31 per week in the previous year (Figure 1b).

**Figure 2:**
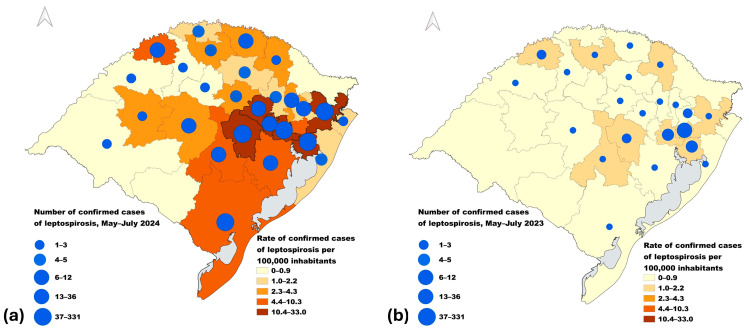
Number of confirmed cases of leptospirosis (circles) and incidence rates (areas) by Health Region, Rio Grande do Sul, Brazil, (**a**) during the flood period (May to 31 July 2024) and (**b**) during the same period in the previous year (1 May to 31 July 2023).

Most of the confirmed cases (78.5%) were detected in six Health Regions (the highest quintile of cases); in these six regions, 50 or more confirmed cases of leptospirosis were reported during the period (Table 1). The number of cases ranged from 0 to 353 across the 30 Health Regions, with a median of 5.5 cases. The region with the highest number of cases was Health Region 10, which includes the state capital (Figure 2). Four out of six regions in the highest quintile of cases were in the Metropolitan macroregion, the most populated area, and home of the main tributary rivers (Cai, Gravatai, Sinos, and Paranhana) of the Guaiba river, which represented the main flooded area. Other Health Regions in the highest quintile of cases included Region 28 in the Valleys Macroregion with the Taquari and Jacui rivers—also major tributaries of the Guaiba—and Region 21 in the South macroregion, located at the southernmost shores of Lagoa dos Patos, which receives water from rivers coming from the north. Compared with the previous year (93 cases; range by Health Region, 0–30), the macroregion with the highest number of cases was also the Metropolitan, followed by the North and Valleys (Table 1 and Figure 2, using the same scale).

The incidence rate of confirmed cases among Health Regions ranged from 0 to 33.0 per 100,000 inhabitants during the flood period. The highest rate was in Health Region 6, in the Metropolitan macroregion. Two other Health Regions in the same macroregion were also in the highest quintile of incidence rates. However, the macroregion with the highest overall incidence rate was the Valleys, in which three out of four Health Regions were in the highest quintile (Table 1 and Figure 2). When comparing all incidence rates with the baseline (range: 0–2.2), most Health Regions fell into the lowest quintiles compared to the previous year (Figure 2).

**Figure 3:**
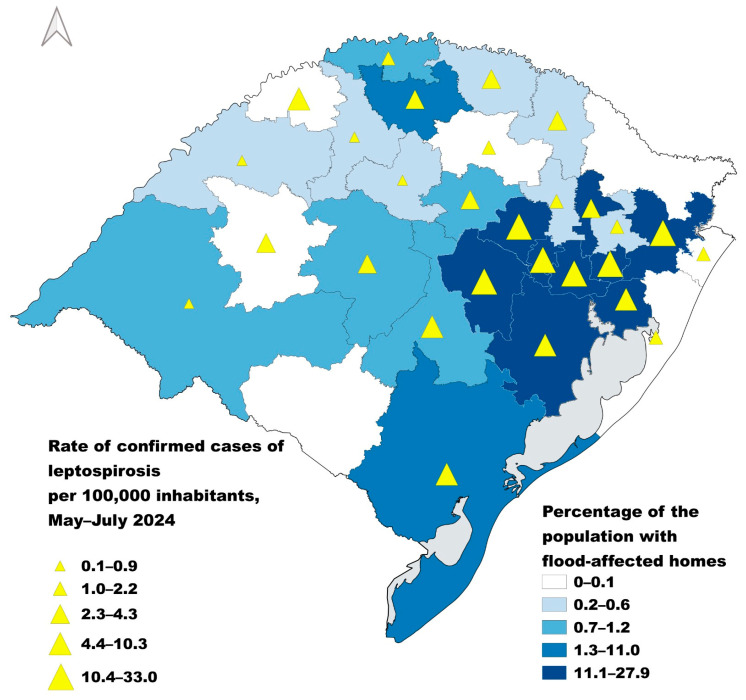
Leptospirosis incidence rate by 100,000 inhabitants during the flood period (1 May to 31 July 2024) over the percentage of the population who had their house flooded in municipalities that were in a situation of public calamity, by Health Region, Rio Grande do Sul, Brazil.

Examining the correlation between the incidence rate of leptospirosis during the flood period and the percentage of the population with flooded households, four out of six Health Regions in the highest quintile of incidence rates were also in the highest quintile for the percentage of the population with flooded households. Spearman’s rho was 0.77, indicating statistical significance (*p*-value < 0.0001) for the correlation between Health Regions ranked by incidence rates of confirmed cases during the flood period and Health Regions ranked by the proportion of the population with flooded households. This indicates a high correlation between geographic areas with households flooded by the disaster and those where the burden of leptospirosis was higher during the flood period (Figure 3).

#### 3.1.2. The Rise in the Number of Cases After the Flood

Comparing the number of confirmed cases during the flood period (1 May to 31 July 2024) with the same months in the previous year used as a baseline, the cases went from 93 cases in 2023 to 958 cases in 2024; this represents a 10.3-fold increase in confirmed leptospirosis cases in the state after the massive flood. Considering the state population, the leptospirosis incidence rate increased from 0.8 per 100,000 inhabitants to 8.5 per 100,000 inhabitants in the 3-month period. The increase in leptospirosis cases by Health Region ranged from no increase to a 36-fold rise (Table 1). The macroregions South and Valleys had greater increases (25.7-fold and 15.8-fold, respectively), followed by Metropolitan (11.9-fold). These were the three macroregions where the highest percentages of the population had flooded households.

#### 3.1.3. Demographic Characteristics of the Confirmed Cases During the Flood Period Compared with the Baseline

Among the confirmed cases during the flood period, 69.0% were males, with no statistical difference from the baseline (68.8%). Most cases were in the productive age group of 20–59 years (80.2%), similar to the baseline (71.0%) (Table 2).

#### 3.1.4. Leptospirosis Confirmed Deaths During the Flood Period Compared with Baseline

**Figure 4:**
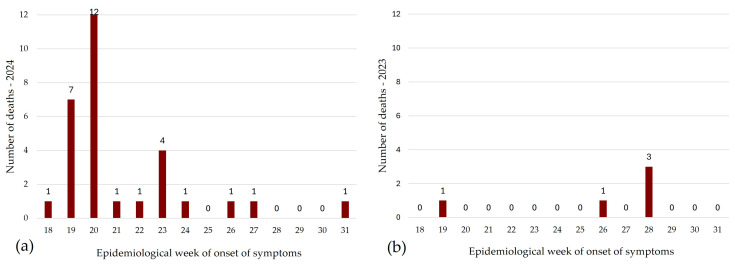
Distribution of confirmed deaths caused by leptospirosis during (**a**) the flood period (1 May to 31 July 2024) and (**b**) the same period in the previous year (1 May to 31 July 2023), Rio Grande do Sul, Brazil.

During the three months of the event, 30 deaths from leptospirosis were confirmed; among them, 3 were females (10.0% of the cases). Most of the deaths were in the productive age group; the age group with the highest percentage was 50–59 years (36.7%). Considering the 958 confirmed cases, the case fatality rate was 3.1%. The fatality rate by age group for individuals 50 years old or over ranged from 7.0% (50–59 years) to 13.3% (70 years or older), higher than that for the younger age groups (ranging from 0 to 1.5%). During the same period in 2023, five deaths were confirmed (one female), two in the 30–39 age group and three in the 60–69 age group (Table 3). Comparing the 5 deaths among the 93 confirmed cases in 2023 (case fatality rate of 5.4%), lethality during the flood was lower. Nevertheless, the increase in the number of deaths was 6.0-fold. Nineteen out of thirty deaths had the onset of symptoms during weeks 19 and 20, one to two weeks after the beginning of the flood (Figure 4).

### 3.2. Main Activities Carried Out During the Response to the Catastrophic Flood Related to Leptospirosis

#### 3.2.1. Risk Communication

Efforts to reduce the likelihood of cases and outbreaks occurring are an essential part of the response. One of the first activities undertaken by the responders, as part of the Crisis Situation Room, was to disseminate information (via radio, television, and social networks) on the prevention and control of leptospirosis to the general public and health professionals. Specific post-flood recommendations included instructions on ensuring the use of safe water; the use of personal protective equipment (PPE) such as gloves and boots for cleaning residual mud in houses and how to clean water; educational materials on the signs and symptoms of leptospirosis and the importance of seeking health care; and attention to the health of workers involved in the response [18,40]. Numerous technical notes and other documents for health professionals were developed and published, such as the Basic Guide to Risks and Care in Floods [41,42]. Despite disseminating numerous official information materials, risk communication remained a challenge on social media during the event, where information was not always accurate.

#### 3.2.2. Revisions to the Use of Antibiotic Chemoprophylaxis During Massive Floods

In the early days of the emergency, another activity undertaken was reviewing existing literature and consulting experts to determine whether antibiotic chemoprophylaxis was recommended for individuals highly exposed during a massive flood. Due to the large number of rescue personnel (military and civilian), responders consulted multiple sources for updated information on the use of antibiotic chemoprophylaxis for high-risk groups in this type of emergency. However, no conclusive evidence was available to support decision-making during a catastrophic flood [43]. After consulting local medical societies and reviewing the literature, which does not recommend mass chemoprophylaxis during climate disasters as a preventive measure for the general affected population, and considering the limited availability of antimicrobial supplies, CEVS recommended chemoprophylaxis in a restricted manner for first responders (military or civilian rescue teams). The lack of conclusive studies in this field posed a challenge for the decision-makers and created an issue in risk communication with the population.

#### 3.2.3. Estimation of the Number of Cases and Distribution of Supplies

Estimating the number of cases by Health Region was essential in the distribution of supplies and in anticipating the number of severe cases expected to require hospitalization. The CEVS team consulted experts in modeling and risk analysis to discuss estimations of the number and location of expected cases. However, developing complex models to estimate the number of cases in different areas within a few days during a disaster proved challenging. Previous publications with endemic risk maps of leptospirosis in the state, along with subarea data from a previous flood event that occurred in the same state (with a much lower magnitude of affected municipalities), were used to estimate the number of expected cases, considering the flood-affected population in each region [15,17,19,21]. The distribution of health supplies was based on the overall population of each Health Region and the number of people affected, with additional supplies sent to regions that included more municipalities identified as higher-risk areas for leptospirosis. The affected municipalities managed the antibiotic stocks, prioritizing the treatment of suspected and confirmed cases over chemoprophylaxis.

#### 3.2.4. Detection and Confirmation of Suspected Cases of Leptospirosis

The early detection of leptospirosis cases can save lives, but conducting surveillance during large-scale floods is challenging. The detection and confirmation of suspected leptospirosis cases was one of the most challenging aspects of the response. Since rapid laboratory tests were unavailable for point-of-care screening, the state public health laboratory organized itself to respond quickly to the large volume of samples. The diagnostic methods used at the Rio Grande do Sul Central Laboratory included real-time polymerase chain reaction (RT-PCR) and serology for IgM antibodies (ELISA test). To perform the Microagglutination Test (MAT), samples were sent to the National Reference Laboratory for Leptospirosis (LRNL)—Fiocruz IOC/RJ, located in the state of Rio de Janeiro, which provided technical support and released results within 24–72 h after receiving the sample. MAT remains the “gold standard” reference method recommended by the Brazilian Ministry of Health and the World Health Organization for confirming the serological diagnosis of leptospirosis [3,18,26,44]. However, it is not performed at the state level, requiring the transportation of biological samples to the national reference laboratory. Other differential diagnoses, based on the patient’s clinical manifestations, were conducted based on the clinical suspicion of cases.

Another major challenge faced by responders and most of the affected communities was the interruption of electricity, telephone, and internet services (the building where the state government’s data center is located was underwater), which made it impossible for health professionals to access the online official surveillance system at the beginning of the event. As an alternative, a parallel brief online form was developed to send data using cellphone devices and implemented in the first week of the event to track six priority diseases, including leptospirosis. The epidemiological surveillance departments of the municipalities and hospital field teams adopted the new provisional form. The state capital implemented its own electronic forms to report suspected cases of leptospirosis. These forms were sent daily to CEVS, and additional monitoring was carried out using laboratory registration data as part of the National Reference Laboratory Network at the state level. This enabled the rapid monitoring of suspected leptospirosis cases that had not yet been registered in the official national notification system (SINAN).

Eventually, SINAN was re-established in the state, allowing for the retrieval of completed case notification forms submitted by municipalities. This integrated surveillance system enabled responders to monitor the epidemiological situation and provide daily epidemiological reports and strategic data to support the Situation Crisis Management Team established by the government and to inform the general population. However, the use of multiple data sources made data management and epidemiological analysis very complex during the disaster.

## 4. Discussion

The 2024 catastrophic flood in Rio Grande do Sul is considered the worst climate disaster in the state’s history, affecting over 2.3 million people [33,35,45]. Given the scale of this event, a surge in leptospirosis cases was anticipated, following patterns observed in similar flood events across the Americas and other regions [2,8,10,46,47,48]. During the event, a total of 958 cases of leptospirosis were identified (a 10.3-fold increase compared to the previous year), and 30 deaths were caused by the disease (a 6.0-fold increase). Understanding the extent of this increase and identifying the most affected areas are critical for decision-makers when planning an effective public health response.

The relationship between rainfall and leptospirosis incidence has been well documented worldwide [4,49,50]. While many studies have reported leptospirosis outbreaks after floods, most lack estimates of the extent of the increase. Among those that have provided estimates, a study in Mumbai, India, found an eightfold increase in incidence post-flood, while another in Kelantan, Malaysia, reported a doubling of cases [6,7]. Furthermore, research in Bahia, Brazil, found that a weekly cumulative rainfall anomaly of 20 mm increased the risk of leptospirosis by 12% compared to typical seasonal patterns [51]. Our study identified a 10.3-fold increase in cases and a 6.0-fold increase in deaths, which generally aligns with these findings.

Rather than focusing solely on rainfall, our study assessed exposure using a different variable: the percentage of the population with flooded households in municipalities that were in a state of public calamity. We observed a strong correlation between the incidence rates of confirmed cases and this proxy measure of risk. Individuals whose homes were flooded likely experienced multiple exposure risks, both during the flood and throughout post-disaster cleanup efforts. The timeline of reported cases was consistent with findings from previous outbreaks [6,48].

Although Brazil’s SUS Health Surveillance System has a well-defined structure and received support from various agencies involved in the response, it was not adequately prepared to address an event of this magnitude. Several critical challenges emerged, including limited communication due to power and internet outages, which impeded access to official information systems; the complete isolation of entire regions due to road blockages, which disrupted the transport of individuals and biological samples; the absence of strategic stockpiles; and logistical difficulties in receiving essential supplies from across the country. To mitigate these challenges, the state health surveillance center, in collaboration with state partners and the Emergency Operations Center (EOC), implemented a series of life-saving public health measures. These included the adoption of a chemoprophylaxis protocol for rescue personnel, the dissemination of guidelines for the early treatment of symptomatic individuals, and the establishment of alternative communication channels to facilitate the reporting of suspected cases of leptospirosis and other high-risk health conditions during the emergency.

In response to these challenges, the government of Rio Grande do Sul developed the Plano Rio Grande for Reconstruction, Adaptation, and Climate Resilience [52], which outlines a comprehensive framework for mitigating the impacts of similar future events. The Plano Rio Grande proposes measures to mitigate the impacts caused by the flood and to expand infrastructure for a better response to emergencies. In health surveillance, strategies have been developed to prepare municipal surveillance teams to reduce health risks.

Managing the confirmation of suspected leptospirosis cases during the disaster posed significant challenges. The number of suspected cases surged from approximately 30 per week in the baseline period to nearly 450 suspected cases per week during the flood. Even with national support, this influx overwhelmed state resources. The unavailability of rapid diagnostic tests at local healthcare facilities at the start of the event, combined with road disruptions that restricted sample transportation to LACEN/RS (located in the highly affected metropolitan area), further complicated case confirmation. As a result, health professionals were instructed to initiate treatment based on clinical suspicion rather than waiting for laboratory confirmation. Future research should focus on identifying the most effective diagnostic tests for emergency situations. The development of inexpensive, rapid diagnostic tools could greatly enhance outbreak response efforts, ensuring timely and accurate case detection.

A limitation of this study is that it relied on case data from a single year (2023) as a baseline. During the COVID-19 pandemic (2020–2022), there was significant underreporting of leptospirosis cases. While the state’s average is approximately 500 cases per year, only an average of 230 cases were reported annually during the three pandemic years. As a result, only 2023 data were used in this study to avoid the impact of underreporting during the pandemic period.

Leptospirosis presents a broad spectrum of symptoms, often making it difficult to distinguish from other acute febrile illnesses [18,38]. During natural disasters, such as floods, many cases remain undiagnosed, as infected individuals may be asymptomatic or experience only mild symptoms. It is estimated that for every symptomatic case, there are five to ten subclinical cases [53]. Following the critical flood period and the subsequent cleaning of flooded houses and buildings, the incidence of leptospirosis returned to the endemic level. In the next three months (August to October, 2024), there were 75 cases confirmed in the state, and two deaths [37].

In addition, the frequent co-circulation of other infectious diseases with overlapping symptoms during these emergencies, such as dengue and influenza, highlights the critical need for a multi-disease differential diagnosis in conjunction with epidemiological monitoring. Regarding the epidemiological situation of dengue in the state of Rio Grande do Sul, the number of cases remained below 50,000 per year until 2022, which could be considered low compared to other states in Brazil. However, when the flood began, the state was already experiencing a dengue epidemic. In epidemiological week 18 alone, 14,864 cases were confirmed, and a total of 172,000 dengue cases were confirmed in 2024 [37]. Due to the high number of dengue cases, a proportion of the samples that tested positive for leptospirosis were also tested for dengue using ELISA-IgM.

Our study identified that 70% of cases occurred among males, with most aged between 20 and 59 years, a demographic profile likely involved in rescue and cleanup activities. This aligns with prior findings [2,6,17,54]. The observed case–fatality rate was 3.1%, lower than Brazil’s national average (9%) and the global average (7%), possibly due to the higher sensitivity of the health system in identifying cases during the emergency [1]. It was closer to the overall case–fatality rate of 5% (ranging from 0% to 60%) found in a systematic review of outbreaks [2]. However, the case–fatality rate increased significantly with age. Among individuals aged 50 and older, the case–fatality rate of 7.8% was six times higher than in younger age groups (1.3%), with a sharp increase between individuals with 70 or more years of age. A study in Colombia, outside of a flood context, reported an 8.5% case–fatality rate with a mean age of 47 years [55]. In the 20-to-49-year age group in Rio Grande do Sul, the case–fatality rate ranged from 1.0% to 2.0%, suggesting that risk communication efforts targeting both the general population and healthcare workers may have contributed to earlier diagnosis and the timely initiation of antibiotic therapy. The overall lower fatality rate observed—compared to other studies—could be attributed to these communication efforts. However, it is also possible that the surveillance system was more sensitive in capturing mild cases, which could explain the lower-than-expected lethality. Regarding the increased fatality rate with age, comorbidities may play a significant role in prognosis. Further research is needed to investigate the specific factors contributing to death from leptospirosis during this flood, including a detailed analysis of individual cases. This should consider preexisting health conditions, leptospirosis symptoms, and the timing of diagnosis and treatment initiation, as well as the quality of in-hospital care.

The potential use of antibiotic chemoprophylaxis for preventing leptospirosis during major floods has been debated within the scientific community. A 2024 systematic review reassessed the available evidence, building on a previous Cochrane review, found uncertainty regarding the impact of certain antibiotics compared to the placebo on leptospirosis mortality, and noted that none of the trials assessed serious adverse effects [43].

Another 2024 study concluded that, despite several methodological limitations, multiple trials evaluating antibiotic chemoprophylaxis suggested a protective effect and that the potential benefits outweighed the risks, given the low rates of adverse effects [56]. A 2017 mathematical modeling study also explored various chemoprophylaxis scenarios and concluded that “evidence of the effectiveness of post-exposure prophylaxis is inconsistent; however, the direction of the association supports a protective effect for morbidity and mortality” [57]. The effectiveness of such interventions may depend on various factors, including the timing and coverage of prophylaxis. Further research is needed to establish clear, evidence-based recommendations for leptospirosis control during floods.

Previous research has identified a higher risk of leptospirosis in metropolitan areas of Porto Alegre, particularly near the Taquari, Cai, and Jacuí rivers, which are tributaries of the Guaíba river [20,21,22]. Our study corroborates these findings, identifying the Metropolitan and Valleys macroregions as high-risk zones. The distribution of cases during the flood underscores the importance of environmental factors previously associated with leptospirosis in Rio Grande do Sul, including ecoregions, soil types, agricultural practices (notably rice cultivation), and low altitudes. Additionally, the presence of major rivers and lagoons in these areas further contributes to the heightened risk, emphasizing the need for targeted surveillance and mitigation strategies in these vulnerable regions.

Socioeconomic and environmental drivers play a crucial role in leptospirosis transmission, underscoring the need for a One Health approach when addressing this disease [21,58,59,60]. Climate change, alongside the growing overlap of habitats for both disease reservoirs and human populations, is expected to exacerbate the incidence, frequency of outbreaks, and mortality rates associated with leptospirosis in the coming years [56,61]. A previous study on leptospirosis in Rio Grande do Sul advocated for the adoption of a One Health approach that acknowledges the intricate interactions between humans, animals, and ecosystems. This approach emphasizes the necessity for a multidisciplinary and intersectoral plan involving public health, civil defense, and agricultural sectors [21].

In a prior publication addressing the catastrophic flood event, the authors recommended a comprehensive strategy for flood response in Rio Grande do Sul, Brazil. This strategy would not only address immediate public health needs but also focus on long-term recovery and the prevention of future outbreaks. The researchers emphasized the importance of effective collaboration among governments, health authorities, policymakers, and local communities to mitigate the disaster’s impact and enhance recovery efforts [45].

Currently, there is no human vaccine for leptospirosis available in Brazil, and only a few countries worldwide offer one, typically targeting specific high-risk groups. While some researchers are actively working on developing new vaccines to combat this significant public health threat, leptospirosis remains a critical issue [28,62]. According to a roadmap developed during an expert meeting, leptospirosis is not yet considered a “tool-ready” disease for global initiatives [29]. As a result, surveillance programs require new strategies and tools, including the development of affordable, rapid diagnostic tests and human vaccines, to enhance early detection and prevention.

Despite its significant public health burden, leptospirosis remains a neglected disease, affecting approximately one million people worldwide each year, with a fatality rate of 7% [1]. It is not currently listed among the World Health Organization’s (WHO) list of neglected diseases, nor is it included in key global initiatives, such as the WHO’s “Ending the Neglect to Attain the Sustainable Development Goals: A Roadmap for Neglected Tropical Diseases” [63], the Pan American Health Organization’s (PAHO) Disease Elimination Initiative [64], or Brazil’s program to eliminate 14 socially determined diseases [65]. This omission underscores leptospirosis’ status as a “silent epidemic”, one that urgently requires global attention and action [58]. Given the escalating environmental and socioeconomic challenges, including the rise in climate-related disasters due to climate change, addressing leptospirosis must be prioritized in both national and international public health agendas.

## 5. Conclusions

This study describes a severe leptospirosis outbreak following the catastrophic flood in Rio Grande do Sul, Brazil, with an estimated 10.3-fold increase in the number of cases. A strong correlation was observed between incidence rates and the percentage of the population with flooded households in municipalities under a state of public calamity. Despite efforts to mitigate the flood’s impact, the outbreak placed an overwhelming burden on the healthcare system, resulting in 30 fatalities during the flood period.

Leptospirosis is a life-threatening disease that remains difficult to control due to its numerous animal reservoirs, diverse serogroups, and multiple environmental drivers. Current preventive measures are insufficient, and urgent action is needed to save lives. Health surveillance plays a crucial role in responding to large-scale flood events by generating real-time data to guide and prioritize interventions. To strengthen preparedness and response efforts for diseases and health risks associated with such emergencies, investments should be directed toward the development and implementation of strategic plans for allocating human resources and essential supplies. Additionally, priority should be given to the creation of adaptive and efficient detection tools, such as rapid local diagnostic tests; the reassessment of diagnostic methods and criteria; and the development of clinical protocols tailored to the specific characteristics of each emergency. Furthermore, a mechanism to report suspected cases when power and internet access are unavailable would be helpful. Further research into antibiotic chemoprophylaxis for rescue personnel could also enhance disease prevention strategies during major flood events. The data and the events presented in this article may provide valuable insights for decision-makers seeking to prevent and manage leptospirosis outbreaks during future climate disasters.

## Figures and Tables

**Table 1:** Health Region, total population, population with flooded households, and number and rate of confirmed leptospirosis cases per 100,000 inhabitants during the flood period (1 May to 31 July 2024), number and rate for the same period in the previous year (1 May to 31 July 2023), and fold increase in the number of cases, Rio Grande do Sul, Brazil.

Macroregion	Health Region	Population	Population with Flooded Households n (%)	2024	2024	2023	2023	Increase 2024/2023
				Total (n)	Total (Rate)	Total (n)	Total (Rate)	(Fold)
	R01	459,477	3221 (0.7)	17	3.7	2	0.4	8.5
	R02	123,416	48 (0.0)	3	2.4	0	0.0	NA
	R03	459,790	4772 (1.0)	2	0.4	0	0.0	NA
(Midwest)		1,042,683	8041 (0.8)	22	2.1	2	0.2	11.0
	R04	158,759	204 (0.1)	2	1.3	0	0.0	NA
	R05	233,267	115 (0.0)	4	1.7	1	0.4	4.0
	R06	233,156	33,020 (14.2)	77 *	33.0 *	3	1.3	25.7
	R07	823,873	128,458 (15.6)	85 *	10.3	11	1.3	7.7
	R08	774,092	216,134 (27.9)	108 *	14.0 *	10	1.3	10.8
	R09	411,012	79,465 (19.3)	36	8.8	1	0.2	36.0
	R010	2,359,108	195,325 (8.3)	353 *	15.0 *	30	1.3	11.8
(Metropolitan)		4,993,267	652,721 (13.1)	665	13.3	56	1.1	11.9
	R011	280,947	633 (0.2)	2	0.7	1	0.4	2.0
	R012	128,372	442 (0.3)	1	0.8	0	0.0	NA
	R013	229,079	466 (0.2)	2	0.9	1	0.4	2.0
	R014	224,705	63 (0.0)	15	6.7	5	2.2	3.0
(Missionary)		863,103	1604 (0.2)	20	2.3	7	0.8	2.9
	R015	183,582	1537 (0.8)	4	2.2	0	0.0	NA
	R016	233,214	1476 (0.6)	6	2.6	2	0.9	3.0
	R017	413,755	605 (0.1)	5	1.2	3	0.7	1.7
	R018	133,018	494 (0.4)	3	2.3	2	1.5	1.5
	R019	117,377	1103 (0.9)	5	4.3	1	0.9	NA
	R020	163,362	3020 (1.8)	5	3.1	3	1.8	1.7
(North)		1,244,308	8235 (0.7)	28	2.3	11	0.9	2.5
	R021	877,265	96,224 (11.0)	77 *	8.8	3	0.3	25.7
	R022	188,139	5 (0.0)	0	0.0	0	0.0	NA
(South)		1,065,404	96,229 (9.0)	77	7.2	3	0.3	25.7
	R023	612,993	1052 (0.2)	7	1.1	4	0.7	1.8
	R024	99,512	3 (0.0)	0	0.0	0	0.0	NA
	R025	314,146	1997 (0.6)	5	1.6	1	0.3	5.0
	R026	187,679	5889 (3.1)	8	4.3	1	0.5	8.0
(Mountains)		1,214,330	8941 (0.7)	20	1.6	6	0.5	3.3
	R027	203,155	2456 (1.2)	21	10.3	3	1.5	7.0
	R028	349,679	26,428 (7.6)	52 *	14.9 *	4	1.1	13.0
	R029	224,513	46,744 (20.8)	31	13.8 *	1	0.4	31.0
	R030	129,163	25,165 (19.5)	22	17.0 *	0	0.0	NA
(Valleys)		906,510	100,793 (11.1)	126	13.9	8	0.9	15.8
State		11,329,605	876,564 (7.7)	958	8.5	93	0.8	10.3

Note: * Highest quintile.

**Table 2:** Distribution of confirmed cases of leptospirosis according to age group and sex during the flood period (1 May to 31 July 2024) and in the same months in the previous year, Rio Grande do Sul, Brazil.

Demographic Characteristics		2024		2023		*p*-Value *
		n (%)	Rate	n (%)	Rate	
Age						0.088
1	0 to 19	74 (7.8)	2.9	9 (9.7)	0.3	
2	20 to 29	204 (21.4)	13.3	18 (19.4)	1.2	
3	30 to 39	202 (21.2)	12.5	23 (24.7)	1.4	
4	40 to 49	205 (21.5)	13.4	20 (21.5)	1.3	
5	50 to 59	157 (16.5)	11.0	5 (5.4)	0.4	
6	60 to 69	82 (8.6)	6.8	14 (15.1)	1.2	
7	70 or more	30 (3.1)	3.0	4 (4.3)	0.4	
	Missing age	4		0		
Sex						0.971
	Female	297 (31.0)	5.3	29 (31.2)	0.5	
	Male	661 (69.0)	12.6	64 (68.8)	1.2	
	Missing sex	0		0		
Total n (%)		958 (100.0)		93 (100.0)		

* *p*-value for the chi-squared test of heterogeneity.

**Table 3:** Distribution of confirmed cases and deaths by leptospirosis according to sex and age groups, and the fatality rate, during the flood period (1 May to 31 July 2024), Rio Grande do Sul, Brazil.

Demographic Characteristics		Cases 2024	Deaths 2024	Fatality Rate (%)
		n (%)	n (%)	
Age				
	0 to 19	74 (7.8)	0 (0)	0
	20 to 29	204 (21.4)	2 (6.7)	1.0
	30 to 39	202 (21.2)	4 (13.3)	2.0
	40 to 49	205 (21.5)	3 (10.0)	1.5
	50 to 59	157 (16.5)	11 (36.7)	7.0
	60 to 69	82 (8.6)	6 (20.0)	7.3
	70 or more	30 (3.1)	4 (13.3)	13.3
	Missing	4	0	
Sex				
	Female	297 (31.0)	3 (10.0)	1.0
	Male	661 (69.0)	27 (90.0)	4.1
	Missing	0	0	
Total n (%)		958 (100.0)	30 (100.0)	3.1

## Data Availability

All the data used can be downloaded, aggregated by municipality, from: Ministry of Health of Brazil, Notifiable Diseases Information System (SINAN). Available online: http://200.198.173.165/scripts/deftohtm.exe?snet/leptorsnet (accessed on 13 March 2025).

## Supplementary Materials

The following supporting information can be downloaded at: https://www.mdpi.com/article/10.3390/pathogens14040393/s1, Figure S1: Map of the 30 health regions and the 7 health macroregions in the state of Rio Grande do Sul, Brazil; Table S1: Criteria for case definitions of leptospirosis according to the Ministry of Health of Brazil; Table S2: Methodological details of the laboratory tests used to confirm leptospirosis cases in Brazil.

## Abbreviations

The following abbreviations are used in this manuscript:

CEVS	The State Health Surveillance Center
OEC	Operational Emergency Center
SINAN	Notifiable Diseases Information System
SUS	Universal Public Healthcare System
