# Supplementary material for: Leptospirosis Cases During the 2024 Catastrophic Flood in Rio Grande Do Sul, Brazil
Source: Pathogens. 2025 Apr 17;14(4):393. doi: 10.3390/pathogens14040393 (PMC12030144; doi:10.3390/pathogens14040393)
Supplement: Supplementary file 1 [file pathogens-14-00393-s001.zip › pathogens-3569267-supplementary.pdf]

Supplementary Materials

Figure S1: Map of the 30 health regions and the 7 health macroregions in the state of Rio Grande do Sul, Brazil

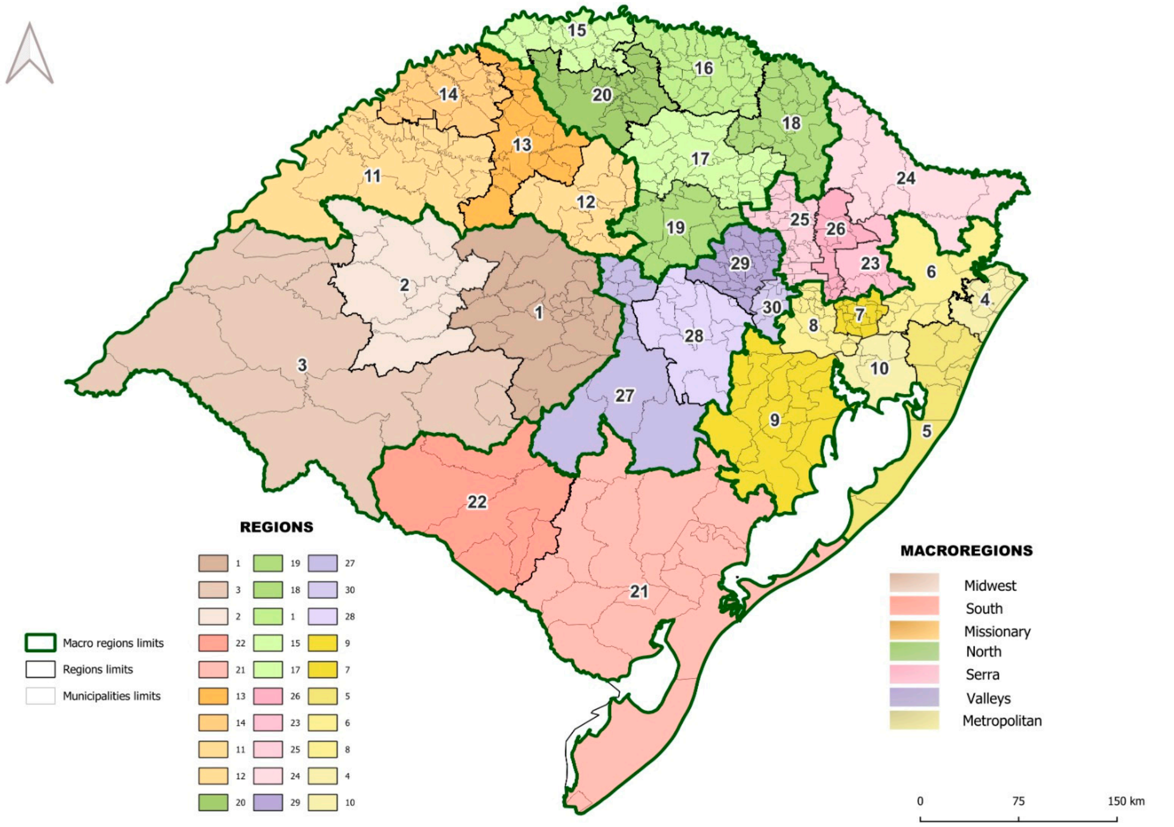

Table S1. Criteria for case definitions of leptospirosis according to the Ministry of Health of Brazil.

Leptospirosis suspected case	An individual presenting with fever, headache and myalgia with either one of criteria listed below. Criteria 1- Presence of suggestive epidemiological history in the 30 days prior to the date of onset of symptoms, such as: a) exposure to flood, mud or water reservoirs; b) exposure to sewage, pits, garbage and rubble; c) activities involving occupational risk, such as garbage collection and material for recycling, cleaning streams, working in water or sewage, handling animals, agriculture in flooded areas; d) epidemiological link to a case confirmed by laboratory criteria; e) living or working in areas at risk for leptospirosis. Criteria 2 - Presence of one or more of the following signs or symptoms: a) jaundice; b) high levels of bilirubin; c) conjunctival suffusion; d) hemorrhagic phenomena; e) signs of acute renal failure.
Leptospirosis confirmed case by clinical-laboratory criteria	Suspected case associated with one or more of the following laboratory test results:<br><br>Result 1: Reagent enzyme-linked immunosorbent assay (ELISA-IgM), plus microagglutination test (MAT) seroconversion with two samples, with a non-reagent first sample (acute phase) and a second sample (14 days after the onset of symptoms with a maximum of up to 60 days) with a titer greater than or equal to 200;<br><br>Result 2: ELISA-IgM reagent, plus four-fold or greater increase in antibody titer by MAT between two blood samples collected, with the second sample between 14 and 60 days of the onset of symptoms;<br><br>Result 3: ELISA-IgM reagent, plus first MAT sample with titer greater than or equal to 800;<br><br>Result 4: Real-time Polymerase chain reaction test (qPCR) detectable result.
Leptospirosis confirmed case by clinical-epidemiological criteria	All suspected cases that present fever and changes in liver, renal or vascular functions, associated with an epidemiological history (described in the definition of a suspected case) and who has a negative result for other diseases and who present one of the following criteria: a) for some reason, has not collected material for specific laboratory tests; b) has a non-reagent result with a single sample collected before the seventh day of illness;
Discarded leptospirosis case:	ELISA IgM tests non-reagent from a blood sample collected after the 7th day of onset of symptoms or without the clinical-epidemiologic criteria

Table S2. Methodological details of the laboratory tests used to confirm leptospirosis cases in Brazil.

Enzyme-linked immunosorbent assay (ELISA-IgM)	Anti-Leptospira antibodies were detected using the commercial Panbio Leptospira IgM ELISA kit (Abbott, USA), following the manufacturer's instructions. The performance characteristics of the kit are as follows:<br><br>Sensitivity: 96.5% (95% CI 87.9 – 99.6%)<br>Specificity: 98.5% (95% CI 95.6 – 99.7%)<br><br>Quality control - Two controls were used following the manufacturer's instructions: one positive and one negative.
Microagglutination test (MAT)	The following serovars were included:<br><br>Icterohaemorrhagiae (RGA), Copenhageni (M20), Canicola (Hond Utrech IV), Grippothyphosa (Moska V), Pomona, Australis (Ballico), Bataviae (Swart), Castellonis (Castellon 3), Cynopteri (3522 C), Javanica (Veldrat Batavia 46), Panama (CZ 214K), Pyrogenes (Salinem), Hardjo (Hardjoprajitno), Sejroe (M84), Patoc (Patoc I), Tarassovi (Perepelitsin), Autumnalis (Akiyami A), Hebdomadis, and Wolffi (3705).
Real-time Polymerase chain reaction test (qPCR)	Leptospira spp. detection was performed using real-time PCR (qPCR) in a duplex reaction that amplifies:<br><br>Target Gene: lipL32 (specific to pathogenic Leptospira spp.)<br><br>Internal Control: the RNASEP1 gene to validate DNA extraction.<br><br>The reaction was carried out using the IBMP MixFit I – MasterMix OneStep (Instituto de Biologia Molecular do Paraná, Brazil) according to the manufacturer's guidelines. Primer and probe sequences, along with the cycling conditions, were adopted from Neris et al. (2023) (https://pmc.ncbi.nlm.nih.gov/articles/PMC10052170/). Since these primer and probe details have been previously published, they have not been reproduced in full in this manuscript. The qPCR assay demonstrated a detection limit of one cell per sample, with an average sensitivity of 68% and specificity of 75% (Valente et al., 2024; Neris et al., 2023). (Valente: https://pubmed.ncbi.nlm.nih.gov/38326762/)<br><br>Quality control - Four internal quality controls were implemented:<br>• A positive control for the RNASEP1 gene.<br>• A positive control for the lipL32 gene.<br>• A negative control for DNA extraction.<br>• A negative control for the qPCR reaction.
